# Oral health knowledge among primary school adolescents: A cross-sectional study from Maasai populated areas in Tanzania

**DOI:** 10.1371/journal.pgph.0005435

**Published:** 2025-12-01

**Authors:** Lutango D. Simangwa, Anne N. Åstrøm, Anders Johansson, Irene Kida Minja, Ann-Katrin Johansson

**Affiliations:** 1 Department of Restorative Dentistry and Prosthodontics – School of Dentistry, University of Namibia, Windhoek, Namibia; 2 Department of Clinical Dentistry – Community Dentistry, Faculty of Medicine, University of Bergen, Bergen, Norway; 3 Department of Clinical Dentistry - Prosthodontics, Faculty of Medicine, University of Bergen, Bergen, Norway; 4 Department of Restorative Dentistry, School of Dentistry, Muhimbili University of Health and Allied Sciences, Dar Es Salaam, Tanzania; 5 Department of Clinical Dentistry - Cariology, Faculty of Medicine, University of Bergen, Bergen, Norway; Institute of Public Health Bengaluru, INDIA

## Abstract

Oral health knowledge (OHK) is vital for maintenance of good oral health. However, poor oral health remains a significant burden for individuals globally. This study aimed to assess OHK by ethnicity and its covariates in terms of sociodemographic, clinical oral health status and dental health care utilization among primary school adolescents in rural Maasai populated areas in Tanzania. Using a two-stage cluster sample design, we randomly selected 23 out of 66 eligible rural schools in Arusha region, where a total of 906 (91.6%) adolescents aged 12–14 years accepted to participate an interview and an oral clinical examination. The interview included questions on socio-demographic factors and oral health related behavior, while clinical examination included oral hygiene status, gingival bleeding, DMFT, dental fluorosis and dental erosion. OHK was assessed by asking six questions whereby the higher the sum-score in the index, the higher the knowledge for the adolescent towards oral health. Good OHK was observed on 65.1% (590/906) of the adolescents, whereby good OHK in non-Maasai and Maasai were 79.5% and 61.4%, respectively. For the whole group of Maasais and non-Maasais, clinical variables (oral hygiene status, gingival bleeding, DMFT, dental fluorosis and dental erosion) were not associated with good OHK. However, for the non-Maasai group, having less dental fluorosis was associated with good OHK. Modified Poisson regression analysis revealed that being a non-Maasai (APR = 1.2, 95% CI: 1.1-1.4), having a mother with higher education level (APR = 1.2, 95% CI: 1.1-1.4) and ever visited a dentist for dental check-up (APR = 1.3, 95% CI: 1.1-1.4) were significant predictors for having good OHK. A slight majority of the adolescents investigated had good OHK which was significantly associated with ethnicity, maternal education level and ever visited for dental check-up. There is a need to improve OHK, and emphasize adoption of oral disease prevention efforts.

## Introduction

Oral health knowledge (OHK) is considered to be an essential prerequisite for performance of an adequate oral health-related behavior and important for maintaining a good oral health [1]. Previous reports have shown that increased OHK leads to better oral health [2]. However, poor oral health remains a significant burden for many individuals globally and especially so in developing countries [1]. In this regard, oral health education plays an important role for improving the overall knowledge that may lead to the adoption of a more favorable oral health related behavior and improved oral health [3]. In fact, numerous oral diseases including for example dental caries, gingivitis, periodontal disease and oral cancer can be prevented by an acceptable lifestyle and behaviour [4,5].

Oral health education may, besides in the direct dental setting, also be provided in other arenas such as hospitals, primary health care centers and schools. About one billion children and adolescents worldwide spend a large part of their daily life at school. Schools are therefore an important learning platform for promoting OHK in order to provide the conditions for a long-term good oral health related behavior and an increased chance for a better oral health later in life [2,6]. However, due to insufficient health education and little preventive measures, the prevalence of morbidity due to oral diseases among students is high [2,7] and their oral health status often poor [8,9]. This may also negatively affect their class room performance and even their success later in life [10].

Besides oral health behaviour, sociodemographic factors also plays a great role as determinants of oral diseases [11]. The sociodemographic factors have also been found to be associated with OHK and include, beside gender, for example individual and parental level of education, ethnicity and socioeconomic status [12–15]. In this regard, individuals with higher level of education, higher economic status and being girls has been found to have better OHK compared to individuals with low education level, low socioeconomic status and being boys. Oral health habits among adolescents’ have been found to be closely related to parental educational level, household incomes and family structure [16]. In addition, proper oral health related behaviours such as good oral hygiene habits and regular dental check-ups may increase the possibility of prevention of oral diseases [12]. Oral health related behaviour is also associated with other factors including both OHK and attitude [17–19]. On the same note, lifestyle changes and child-rearing practices can have a significant impact on the oral health of adolescents [20].

In Tanzania, oral health education has been part of the primary school curriculum since 1982 and usually implemented by teachers at schools [21]. However, there are no reports regarding OHK among the primary school students. Reports among secondary school students in Tanzania, 13–17 year old, (2004) showed, however, that only a few of them had good knowledge about gingivitis [22].

As regard oral health in Tanzania our own previous study among primary school students 12–17 year old (2018) from pastoral areas in Tanzania, found 65.6% and 40.9% of adolescents had poor oral hygiene and gingivitis, respectively [8]. Another Tanzanian study among 7–22 year old (2008), found that the prevalence of poor oral hygiene was as high as 73.5% [23]. According to Tanzania National oral health survey report (2020), 33.1% of children aged 5- to 15-year-olds had dental caries and more commonly among rural (38.4%) than urban children (29.2%). Gingivitis affected 57.4% of the children aged 12 and 15 years also more common among rural children (65.2%) than urban (52.0%) [24]. In the literature, it is argued that healthcare utilization should correlate with the need for care, awareness of that need, desire for care, and accessibility [25]. Thus, ensuring adequate access and availability for oral health care services is important. In conjunction with dental care service utilization, the previous National oral health survey report in Tanzania revealed that, 20.4% of the children ever visited a dental clinic, while only 7.3% had visit a dental clinic in the past 12 months with 5.9% visiting due to pain and only 0.5% visited for dental check-up. Dental visits were reported more often among urban (23.1%) than rural (16.4%) children [24].

There is no published literature on OHK among adolescents living in Maasai populated areas in Arusha region, northern part of Tanzania. This study aimed to assess OHK by ethnicity and its covariates in terms of sociodemographic, clinical oral health status and dental health care utilization among primary school adolescents in rural Maasai populated areas in Tanzania. status.

## Materials and methods

### Sampling technique and sample size

This cross-sectional study was conducted among the rural public primary school adolescents in Maasai populated areas of Monduli and Longido districts in Northern part of Tanzania from 03.06.2016 to 30.11.2016. The study aimed to focus on 12–14-year-old adolescents. A total of 100 primary schools were initially listed from both districts. After excluding urban and private schools a total of 66 rural public primary schools were eligible for the study (38 from Monduli and 28 from Longido district). Out of the 66 eligible schools, 23 schools (13 from Monduli and 10 from Longido) were randomly selected using a two-stage cluster sample design with school as the primary sampling unit. From each randomly selected school, classes (6^th^ grade) which was expected to contain adolescents aged 12–14 years were approached and all students in each selected class were invited to participate. A minimum sample size of 845 was estimated based on the assumption that the prevalence of adolescents with good OHK would be 50%, a margin error of 5% and confidence intervals of 95%. Details of the sampling procedure has been described elsewhere [8].

### Interviews

A questionnaire was initially constructed in English and translated into Swahili, the Tanzania national language, by a qualified translator from the University of Dar Es Salaam, Tanzania. Both closed- and open-ended questions were used.

The questionnaire was pre-tested in 50 primary school children, 12–14-year old, before the actual study and modified as needed. The participants in the pilot study were not part of the main study. A face-to-face interview in Swahili with the students were performed by two trained medical nurses [8]. The interview was done in a school setting and each student was interviewed privately while the others were in their classroom.

Socio-demographic factors were assessed in terms of age, ethnicity, gender, place of residence (e.g., living in Monduli or Longido districts), father’s and mother’s level of education, household socio-economic status (perceived affluence of my household and household wealth index) [26]. Ethnicity was assessed by asking: “*What is your ethnic group?”* The response categories were (1) = Maasai, (2) = Meru, (3) = Arusha and (4) = others. During analysis, the items were dichotomized to 0 = Maasai (including option (1)) and 1 = non-Maasai (including option (2), (3) and (4). Parents’ level of education was assessed by asking: “*What is the highest level of school your mother/father has attended?”* Responses were (0) for none, (1) for she/he started but did not complete primary school, (2) for completed primary school (3) for she/he started but did not complete secondary school, (4) for she/he completed secondary school, (5) for she/he started but did not complete college/university, (6) for completed college/university and (7) for I don’t know. In the statistical analyses, the items were dichotomized as (0) for low education (from options (0), (1), (2), (3) and (7) and (1) for high education (from options (4), (5) and (6). Durable household assets indicative of family wealth (i.e., radio, television, refrigerator, mobile telephone, cupboard, bicycle and motorcycle) was recorded as (“yes”) available and in working condition or (“no”) not available and/or not in working condition.

Oral health related behaviors were assessed in terms of dental care service utilization by asking: *“Before today, have you ever visited a dentist/dental therapist for toothache” and “Before today, have you ever visited a dentist/dental therapist for checkup”?* The alternatives for responses were *“yes”* or *“no”*.

A number of clinical variables were recorded in a clinical examination performer by one main investigator (LS). These were gingival bleeding, DMFT, dental fluorosis, dental erosion, tooth wear and temporomandibular disorders (TMD) and clinical procedures and findings have previously been published [8].

OHK was assessed by asking a total of statements for example: *1. “Not doing tooth cleaning properly might cause tooth decay”. 2. “Frequent intake of sugary foods can cause tooth decay” and others.* The response options were “*yes”, “no*” or “*I don’t know*”. The adolescent’s answer was considered as correct or incorrect with a hidden “*I don’t know*” response category. The knowledge of the adolescent was constructed as a sum score of the six statements. Each statement was coded as 1 (correct) and 0 (incorrect). The *“I don’t know”* option was grouped into the incorrect responses. During analysis the knowledge of the adolescent was constructed as a sum score of the six statements (maximum 6 points). The higher score in this sum index the higher the knowledge for the adolescent towards OHK. The sum scores were dichotomized into 0 = poor knowledge and 1 = good knowledge, scoring 50% (3/6) or above and less than 50% of the total score, respectively.

### Statistical analysis

Data entry and processing was carried out using the Statistical Package for Social Sciences (SPSS) for PC, version 25 (IBM corporation, Armonk, NY, USA). STATA 15.0 (Stata corporation, Lakeway drive college station, Texas, USA) was used to adjust for the cluster effect of the primary sampling unit, school. Principle component analysis was used to construct a socio-economic index categorized into wealth quintiles (1^st^ quartile, 2^nd^ quartile, 3^rd^ quartile and 4^th^ quartile implying the poorest, poorer, less poor and least poor respectively) and based on ownership of assets such as furniture and household characteristics including electricity, type of water source, roof material and toilet types. For the purpose of bivariate analysis the four groups were grouped into two groups, most poor (including poorest and poorer) and least poor (including less poor and least poor) [26]. Descriptive statistics were carried out followed by bivariate analysis using cross tabulations and Pearson’s chi-square statistical test.

Modified Poisson regression analysis (prevalence ratio and 95% CI) was used to determine the socio-demographic, behavioral and clinical oral health covariates for oral health knowledge for the total study group and separately for the two ethnic groups - Maasai and non Maasai. All covariates that were statistically significantly associated with OHK in Pearson’s chi-square test (unadjusted analysis; p < 0.05) were included in the model simultaneously.

### Ethics approval and consent to participate

Ethical clearance was obtained prior to study from the region committees for medical and health research ethics in Norway (REK VEST, reference number **2015/2477)** and the Medical Research Coordinating Committee of Ministry of Health and Social Welfare in Tanzania (reference number **NIMR/HQ/R.8a/VOL.IX/2214)**. Permission to work with primary school children/adolescents was obtained from Ministry of Education and Vocational Training through Monduli and Longido district councils and their respective educational authorities. Participation was voluntary and without compensation. Prior to the participation, informed signed consent was obtained by all participants and their parents. All methods were carried out in accordance with relevant guidelines and regulations. If needed relevant advice and/or referral to the district hospital were given free of charge.

### Inclusivity in global research

Additional information regarding the ethical, cultural, and scientific considerations specific to inclusivity in global research is included in the Supporting Information (S1 Checklist).

## Results

### Sample characteristics

The study included a total of 906 primary school adolescents aged 12–17-year-old (mean age 13.4, SD 1.2) giving a response rate of 91.6%. Initially, a total of 989 adolescents were invited to participate, out of which 59 (6.0%) were absent during interviewing and 24 (2.4%) were excluded due to too high or low age. About 56.1% of the study participants were females. Out of 906 adolescents, 52.9% were from Monduli district and the remaining were from Longido district. Among the participants, 721 (79.6%) were from the Maasai ethnic group and 185 (20.4%) from the non-Maasai ethnic group (the Arusha, Meru, Rangi, Sukuma etc). Details of sociodemographic distribution are presented in Table 1 and has also been described elsewhere [8].

**Table 1:** Frequency distribution of sociodemographic features in the total sample, N = 906 and by ethnic groups, Maasai n = 721, non-Maasai n = 185.

Variable	Total group	Maasai % (n)	Non-Maasai % (n)	p-value
**District** Monduli Longido	52.9 (479) 47.1 (427)	58.0 (418) 42.0 (303)	33.0 (61) 67.0 (124)	<0.001*
**Sex** Male Female	43.9 (398) 56.1 (508)	43.1 (311) 56.9 (410)	47.0 (87) 53.0 (98)	0.341
**Age group** 12-14 years 15-17 years	87.3 (777) 12.7 (113)	86.5 (610) 13.5 (95)	90.3 (167) 9.7 (18)	0.173
**Wealth index** Most poor Least poor	48.8 (438) 51.2 (459)	57.3 (408) 42.7 (304)	16.2 (30) 83.8 (155)	<0.001
**Mother’s education** Low (≤ primary school) High (≥ secondary school)	95.0 (861) 5.0 (45)	96.8 (698) 3.2 (23)	88.1 (163) 11.9 (22)	<0.001*
**Father’s education** Low (≤ primary school) High (≥ secondary school)	92.1 (834) 7.9 (72)	95.0 (685) 5.0 (36)	80.5 (149) 19.5 (36)	<0.001*

*Significant Pearson’s Chi-square test (p < 0.05)

### Oral health knowledge according to oral diseases/conditions (OHK)

A total of 65.1% (590/906) of the adolescents had a good OHK, scoring correctly from 3 to 6 out of 6 statements. Table 2 summarizes the responses of the adolescents regarding the specific questions measuring OHK by ethnic groups. As depicted in Table 2, there was a statistically significant differences in terms of OHK between the Maasais and non-Maasais ethnic groups, especially regarding the causes of tooth decay, how to prevent gingival bleeding, and causes of tooth wear. Prevalence of clinical variables were poor oral hygiene (65.5), gingival bleeding (40.9), dental caries experience (8.8), dental fluorosis TF grade 5–9 (48.6), dental erosion (into dentin) (1.9), tooth wear (into dentin) (16.5) and TMD (11.8) [8].

**Table 2:** Responses to six specific statements regarding causes of oral diseases by ethnicity.

Question	Responses				
	Maasais		Non-Maasais		
Do you know that:	Correct n (%)	Incorrect n (%)	Correct n (%)	Incorrect n (%)	p-value
Not doing tooth cleaning properly might cause tooth decay?	572 (79.3)	149 (20.7)	171 (92.4)	14 (7.6)*	< 0.001
Frequent intake of sugary foods can cause tooth decay	589 (81.7)	132 (18.3)	168 (90.8)	17 (9.2)*	0.004
Frequent intake of sugary drinks can cause tooth decay	531 (73.6)	190 (26.4)	145 (78.4)	40 (21.6)	0.221
Cleaning teeth can prevent your gums from bleeding	425 (58.9)	296 (41.1)	134 (72.4)	51 (27.6)*	0.001
High fluoride concentration in drinking water may cause dental fluorosis	324 (44.9)	397 (55.1)	73 (39.5)	112 (60.5)	0.209
Soft drinks and juices can cause tooth wear	312 (43.3)	409 (56.7)	107 (57.8)	78 (42.2)*	0.001

* Significant Pearson’s Chi-square test

Frequency distribution of OHK (>50%) by sociodemographic-, behavioral- and clinical oral health characteristics in total and ethnic group are shown in Table 3. Oral health knowledge varied statistically significantly according to ethnicity, wealth index, maternal education, and whether an adolescent has ever visited a dentist for checkup before. Good OHK was more common among the non-Maasai adolescents (79.5%) than the Maasai adolescents (61.4%), (p < 0.05). A total of 61.6% of the adolescents from most poor families and 68.4% of the adolescents from least poor families had good OHK (p < 0.05). In addition, 64.0% of the adolescents from families where the mother had low education and 86.7% of the adolescents from families where the mother had high education had good OHK (p < 0.05). For more details on OHK distribution by sociodemographic features and clinical variables, see Table 3. For the total group, good OHK was not associated with better clinical status. Among non-Masais, the OHK varied according to district of residency, wealth index and severity of dental fluorosis where more adolescents from Longido district, least poor families and adolescents with lower TF scores had good OHK compared to their counterparts from Monduli district, most poor families and with high TF scores, respectively, (P < 0.05). Among the Maasai ethnic group, OHK varied only with the district of residency, where more adolescents from Monduli district had good OHK compared to their counterparts from Longido district (Table 3).

**Table 3:** Frequency distribution of good OHK according to oral clinical- and sociodemographic and behavioural characteristics in the total sample (n = 906) and separately among Maasais and non-Maasais.

Variable	Good knowledge % (n)	P-value	Ethnic Maasai % (n)	P-value	Groups Non-Maasai % (n)	P-value
**District of residence** Monduli Longido	66.8 (320) 63.2 (270)	0.260	67.0 (280) 53.8 (163)*	<0.001	65.6 (40) 86.3 (107)*	0.002
**Sex** Male Female	63.6 (253) 66.3 (337)	0.385	60.1 (187) 64.2 (256)	0.580	75.9 (66) 82.7 (81)	0.338
**Age** 12-14 years 15-17 years	65.4 (508) 67.3 (76)	0.695	61.3 (374) 66.3 (63)	0.412	80.2 (134) 72.2 (13)	0.622
**Ethnicity** Maasai Non-maasai	61.4 (443) 79.5 (147)*	<0.001				
**Wealth index** Poorest Least poor	61.6 (270) 68.4 (314)*	0.034	61.8 (252) 60.9 (185)	0.866	60.0 (18) 83.2 (129)*	0.008
**Mother’s education** Low (≤ primary school) High (≥ secondary school)	64.0 (551) 86.7 (39)*	0.002	60.9 (425) 78.3 (18)	0.143	77.3 (126) 95.5 (21)	0.090
**Ever visited a dentist for toothache?** No Yes	64.7 (558) 72.7 (32)	0.278	61.1 (421) 68.8 (22)	0.495	79.2 (137) 83.3 (10)	1.000
**Ever visited a dentist for checkup?** No Yes	64.2 (555) 83.3 (35)*	0.011	60.6 (419) 80.0 (24)	0.052	78.6 (136) 91.7 (11)	0.476
**Oral hygiene status** Poor Good	63.0 (374) 69.2 (216)	0.071	60.1 (297) 64.3 (146)	0.321	77.0 (77) 82.4 (70)	0.474
**Gingival bleeding** No Yes	67.3 (360) 62.0 (230)	0.116	62.6 (251) 60.0 (192)	0.526	81.3 (109) 74.5 (38)	0.410
**DMFT** DMFT = 0 DMFT > 0	64.8 (535) 68.5 (55)	0.555	61.7 (412) 58.5 (31)	0.755	77.8 (123) 88.9 (24)	0.292
**Dental fluorosis** TF score 0–4 TF score 5–9	65.0 (303) 65.2 (287)	1.000	58.3 (201) 64.4 (242)	0.109	84.3 (102) 70.3 (45)*	0.041
**Dental erosion** Grade 0 Grade > 0	63.6 (402) 68.6 (188)	0.169	60.8 (321) 63.2 (122)	0.614	77.9 (81) 81.5 (66)	0.548
**Tooth wear** Grade 0 Grade > 0	62.8 (307) 67.9 (283)	0.126	59.9 (238) 63.3 (205)	0.404	75.0 (69) 83.9 (78)	0.190
**TMD pain** 2Q/TMD = 0 2Q/TMD > 0	65.6 (524) 61.7 (66)	0.492	62.0 (397) 56.8 (46)	0.428	79.9 (127) 76.9 (20)	0.933

*Significant Pearson’s Chi-square test

Socio-demographic and dental care utilization variables found statistically significantly associated with OHK in unadjusted analysis (Table 3) were further analyzed in multiple variable analyses using a modified Poisson regression model. As shown in Table 4, the non-Maasai ethnic group was significantly more likely to have good OHK compared to their counterparts in the Maasai ethnic group (APR = 1.2, 95% CI: 1.1-1.4). Similarly, mothers’ education level was a significant covariate of good OHK (APR = 1.2, 95% CI: 1.1-1.4) compared to adolescents from mothers with low education in addition to ever visited a dentist for checkup (APR = 1.3, 95% CI 1.1-1.4). Stratified by ethnic groups, ever visited a dentist for checkup was significant among the Maasais only, where those who ever visited a dentist had better OHK (APR = 2.6, 95% CI: 1.1-6.5). The wealth index was significant among the non-Maasais only, where participants from least poor families had better OHK compared to participants from most poor families (APR = 2.9, 95% CI: 1.2-6.8) (Table 4).

**Table 4:** OHK regressed on socio-demographic, dental health care utilization variables. In the total study group and stratified by ethnic group. Adjusted prevalence ratios (APR) and 95% confidence intervals (CI).

Dependent variable	Independent variables	APR (95% CI)	Ethnic Maasai APR (95% CI)	Groups Non-Maasai APR (95% CI)
**Dental knowledge** (good knowledge)	**Ethnicity** Maasai Non-maasai	1 1.2 (1.1-1.4)^a^		
	**Wealth index** Most poor Least poor	1 1.0 (0.9-1.1)	1 0.9 (0.7-1.3)	1 2.9 (1.2-6.8)^a^
	**Mother’s education** Low (≤ primary school) High (≥ secondary school)	1 1.2 (1.1-1.4) ^a^	1 2.3 (0.9-6.4)	1 5.1 (0.7-39.5)
	**Ever visited a dentist for checkup** No Yes	1 1.3 (1.1-1.4)^a^	1 2.6 (1.1-6.5)^a^	1 2.1 (0.3-17.1)
	**Dental fluorosis** TF score 0–4 TF score 5–9	1 1.1 (0.9-1.3)	1 1.1 (0.9-1.3)	1 0.9 (0.6-1.3)

^a^ Statistically significant

## Discussion

The present study is, to our knowledge, the first study reporting the level of oral health related knowledge and its covariates in terms of sociodemographic behavior and oral health care utilization among primary school adolescents in Maasai populated areas in Tanzania. In this study, the slight majority of adolescents had a good knowledge on oral health, scoring 50% or more of the total score of six and was significantly associated with ethnicity, mother’s education and visiting a dentist for checkup.

The findings obtained in the present study are similar to those obtained in other studies analysing the relationship between OHK and ethnicity, maternal educational levels and regular dental visit habits [27–29]. Regarding levels of OHK in relation to ethnicity, more non-Maasais were found to have higher levels of knowledge than Maasais adolescents. Up to date there is no study which have compared the OHK of Maasais and non-Maasais in Tanzania, but, varying levels of OHK have been observed in various studies assessing oral health and race/ethnicity in the world. Studies from USA found that Caucasians had a better OHK compared to other racial/ethnic groups [30,31]. The difference in OHK between the two ethnic groups in this study might be contributed by the influence of the cultural differences between them. The Maasais are more disadvantaged compared to the non-Maasais because they tend to live in relatively remote areas, and they have a relatively poor command of Swahili, the national language of Tanzania [32]. Also, the traditional Maasai community have managed to maintain their traditional culture and customs despite the influence of western values. Previous reports have revealed that one of the barriers to access oral health care is a person’s culture and/or environment, which significantly influences behavior which in turn can affect knowledge [33]. In addition, another previous report argued that, low uptake of the formalised healthcare system among Maasai people is linked to blame for their ‘culture’ and for health services not being aligned with their social norms [34]. Thus, in the current study, cultural and/or environmental and economic factors might be reducing their opportunities for obtaining good oral health services and thus affecting their OHK. On the other hand, our own previous study revealed that non-Maasais were more likely to have high intake of biscuits, drinking carbonated soft drinks, fruit juices, tea with sugar, high frequency of cleaning teeth and using toothpaste, as well as low frequency of drinking milk compared to Maasai adolescents [35]. In terms of oral diseases, non-Maasais were less likely to have gingival bleeding but more likely to have dental caries and dental erosion [8]. This reflects that, having better OHK as reported in this study is not necessarily translated into favourable oral health related behaviours as reported in another similar study [36].

In this study it was found that as maternal educational level increased, the level of OHK among the adolescents also increased. This finding is similar to reports obtained in other studies from China (2020) [37]. On the other hand, it is in contrast to another study in Ecuador (2022) which found that years of maternal education was not predictive of increased level of OHK, but did predict both some healthier and unhealthier practices [38]. Findings from the current study can be explained by the role of the maternal education in taking care of their children. Reports from previous studies has shown that maternal higher educational attainment is associated with better health outcomes for their children [39]. This is because parents with higher education level are more likely to have the resources (economy), knowledge, as well as access to health care services which are important in promoting optimal health for their children [39,40]. Parents with higher levels of education might have good skills to effectively communicate with their children about the risks associated with certain behaviours and provide support to them to stay safe and healthy. Eventually, this can lead to better health, knowledge and well-being for their children.

In the current study, although only 5% of adolescents reported to have ever visited a dentist for check-up, the association between OHK and ever visited a dentist for check-up was revealed. Similar findings have been reported from other studies [41,42]. Previous reports have found that regular dental visits gives opportunities for early diagnosis and treatment of oral diseases, thereby having a possibility to reduce dental problems [41]. This is so because, during regular dental visits, dentists may raise awareness about oral diseases prevention and treatment which may explain the association between OHK and ever visited a dentist for check-up as observed in the current study. This study as reported in other previous studies [42,43], gives light on the importance of regular dental attendance. Dental visits for check-up play a great role in promoting awareness and OHK among adolescents, because dental professionals take the opportunity to give oral health education to the patient. In addition, through dental visits, preventive dental services can be provided, which can lead to reduced burden of oral diseases and thereby increasing the oral health related quality of life. Since children and adolescents are prone to great oral health inequalities and high burden of oral diseases, measures are needed to facilitate OHK, availability, and accessibility of dental services for children, especially so, in rural areas. In this regard, oral health education should be integrated into general health education as there are common risk factors linking oral and systemic diseases [44].

Although this study show that many adolescents had good OHK, our own previous study from the same participants revealed that many of them were affected by oral diseases like gingivitis [8]. None of the clinical variables, except for dental fluorosis among the non-Maasais, were significantly different in those who reported good knowledge compared to those who had lesser knowledge. This finding implies that a good OHK does not necessarily result in a better oral health status. In addition, and assessed in terms of ethnicity, the better OHK in the non-Maasais compared to the Maasais did not result in a more favorable oral health status. This implies that having good knowledge on oral health does not necessarily mean practising it. Therefore, apart from knowledge they have, measures need to be taken so they also really implement what they learned about how to prevent oral diseases. In terms of dental caries, the prevalence was so low, especially among the Maasai compared to non-Maasais adolescents, and this might be due to their use of more traditional foods which consist of more proteins and less sugary diets [45]. Another factor for low dental caries rate might be due to poor access to cariogenic foods in the school arena. There were no foods which were being sold by vendors in the school premises in all schools we visited. In the future, if the Maasais in this population will adapt to modern dietary habits, the risk will increase for a number of oral diseases like dental caries and dental erosion. Subsequently, the low prevalence of caries and erosion is more related to their intake of more traditional diet and poor economic status than good OHK.

In this study, the knowledge scores were dichotomized into poor and good based on a 50% cut off point in line with another knowledge study [46]. Another reason for using a 50% cut off point for good knowledge was by considering that majority of adolescents in rural areas would have no access to oral health care services and thus, were more likely not to have received oral health related information from numerous information sources.

### Strengths and limitations

The present study contributes to the literature by assessing the relationships between OHK and ethnicity, level of maternal education, as well as dental visits for check-up. In addition, the study findings provide the opportunity for further investigation to establish a causal relationship between OHK versus ethnicity, maternal knowledge and dental visits plus other additional factors by using a cohort study design. The sample size was adequate and achieved a high response rate (91.6%). In research, the larger the sample size the greater the accuracy of the estimates and also it helps to identify outliers in data and provide smaller margins of error. The following statistical models for binary outcomes are suggested in the literature, binary logistic regression model, modified Poisson regression model and log-binomial regression model depending on some definitive features of the outcome variable [47,48]. Nevertheless, modified Poisson regression model, which was used in this study, is most preferred in cross-sectional studies, especially if the outcome of interest is not rare because it estimates the risk ratios or relative ratios better than binary logistic regression model [47,49]. But, using one examiner only, may introduce observer effect bias and confirmation bias. In observer effect bias, the observer may influence the participants of the study and in confirmation bias, the experimenter interprets the results incorrectly looking for information which conforms to their hypothesis and disregard information that argues against it. However, this effect was reduced by training the investigator prior to beginning of data collection. Another limitation of the present study is that, its cross-sectional design was not able to establish causality. The survey responses were susceptible to self-report bias during questionnaire data collection. Self-report information can be subjected to biases such as recall- and social desirability bias, which may affect the results validity. The cut-off points of 50% for OHK used in this study might have increased the number of adolescents having good OHK. However, this cut off was proposed based on previous similar studies for being straight forward [50].

## Conclusion

The study found that a slight majority of adolescents were having a good oral health knowledge and it was significantly associated with ethnic groups, maternal education and ever visits to the dentist for check-up but not to a better oral health clinical status. Therefore, apart from the need to enhance OHK among the adolescents in Arusha region, measures need to be taken so that they really implement what they learned about how to prevent oral diseases. Our findings can be utilized by policy makers in the planning of oral health promotion programs in public primary schools in Tanzania in order to further improve oral health of adolescents in Maasai populated areas.

## Supporting information

S1 Checklist Inclusivity in global research. (DOCX)

## References

[1] Ashley FP. Role of dental health education in preventive dentistry. In: Murray JJ, editor. Prevention of dental disease. Oxford: Oxford University Press. 1996: 406–14.

[2] Haque SE, Rahman M, Itsuko K, Mutahara M, Kayako S, Tsutsumi A, et al. Effect of a school-based oral health education in preventing untreated dental caries and increasing knowledge, attitude, and practices among adolescents in Bangladesh. BMC Oral Health. 2016;16:44. doi: 10.1186/s12903-016-0202-3 27016080 PMC4807560

[3] Nakre PD, Harikiran AG. Effectiveness of oral health education programs: A systematic review. J Int Soc Prev Community Dent. 2013;3(2):103–15. doi: 10.4103/2231-0762.127810 24778989 PMC4000911

[4] Sheiham A. Dietary effects on dental diseases. Public Health Nutr. 2001;4(2B):569–91. doi: 10.1079/phn2001142 11683551

[5] Sham ASK, Cheung LK, Jin LJ, Corbet EF. The effects of tobacco use on oral health. Hong Kong Med J. 2003;9(4):271–7. 12904615

[6] Jürgensen N, Petersen PE. Promoting oral health of children through schools--results from a WHO global survey 2012. Community Dent Health. 2013;30(4):204–18. 24575523

[7] Gift HC, Reisine ST, Larach DC. The social impact of dental problems and visits. Am J Public Health. 1992;82(12):1663–8. doi: 10.2105/ajph.82.12.1663 1456343 PMC1694558

[8] Simangwa LD, Åstrøm AN, Johansson A, Minja IK, Johansson A-K. Oral diseases and socio-demographic factors in adolescents living in Maasai population areas of Tanzania: a cross-sectional study. BMC Oral Health. 2018;18(1):200. doi: 10.1186/s12903-018-0664-6 30514291 PMC6278057

[9] Mbawalla HS, Masalu JR, Astrøm AN. Socio-demographic and behavioural correlates of oral hygiene status and oral health related quality of life, the Limpopo-Arusha school health project (LASH): a cross-sectional study. BMC Pediatr. 2010;10:87. doi: 10.1186/1471-2431-10-87 21118499 PMC3001697

[10] Haque SE, Rahman M, Itsuko K, Mutahara M, Kayako S, Tsutsumi A, et al. Effect of a school-based oral health education in preventing untreated dental caries and increasing knowledge, attitude, and practices among adolescents in Bangladesh. BMC Oral Health. 2016;16:44. doi: 10.1186/s12903-016-0202-3 27016080 PMC4807560

[11] Lawal FB, Oke GA. Clinical and sociodemographic factors associated with oral health knowledge, attitude, and practices of adolescents in Nigeria. SAGE Open Med. 2020;8. doi: 10.1177/2050312120951066 32922786 PMC7446260

[12] Mizutani S, Ekuni D, Furuta M, Tomofuji T, Irie K, Azuma T, et al. Effects of self-efficacy on oral health behaviours and gingival health in university students aged 18- or 19-years-old. J Clin Periodontol. 2012;39(9):844–9. doi: 10.1111/j.1600-051X.2012.01919.x 22780323

[13] Paulander J, Axelsson P, Lindhe J. Association between level of education and oral health status in 35-, 50-, 65- and 75-year-olds. J Clin Periodontol. 2003;30(8):697–704. doi: 10.1034/j.1600-051x.2003.00357.x 12887338

[14] Cronin AJ, Claffey N, Stassen LF. Who is at risk? Periodontal disease risk analysis made accessible for the general dental practitioner. Br Dent J. 2008;205(3):131–7. doi: 10.1038/sj.bdj.2008.653 18690185

[15] Abu-Gharbieh E, Saddik B, El-Faramawi M, Hamidi S, Basheti M, Basheti M. Oral Health Knowledge and Behavior among Adults in the United Arab Emirates. Biomed Res Int. 2019;2019:7568679. doi: 10.1155/2019/7568679 30881996 PMC6381549

[16] Levin KA, Currie C. Adolescent toothbrushing and the home environment: sociodemographic factors, family relationships and mealtime routines and disorganisation. Community Dent Oral Epidemiol. 2010;38(1):10–8. doi: 10.1111/j.1600-0528.2009.00509.x 19845713

[17] Deinzer R, Micheelis W, Granrath N, Hoffmann T. More to learn about: periodontitis-related knowledge and its relationship with periodontal health behaviour. J Clin Periodontol. 2009;36(9):756–64. doi: 10.1111/j.1600-051X.2009.01452.x 19659893

[18] Harada S, Akhter R, Kurita K, Mori M, Hoshikoshi M, Tamashiro H, et al. Relationships between lifestyle and dental health behaviors in a rural population in Japan. Community Dent Oral Epidemiol. 2005;33(1):17–24. doi: 10.1111/j.1600-0528.2004.00189.x 15642043

[19] Sakki TK, Knuuttila ML, Anttila SS. Lifestyle, gender and occupational status as determinants of dental health behavior. J Clin Periodontol. 1998;25(7):566–70. doi: 10.1111/j.1600-051x.1998.tb02489.x 9696257

[20] Yang R, Tang T, Wu S, Wu L, Lei L, Li H. Self-reported oral health habits, knowledge and conditions of schoolchildren and adolescents in mainland China. J Clin Pediatr Dent. 2023;47(3):96–102. doi: 10.22514/jocpd.2023.017 37143427

[21] Ministry of Health and Social Welfare. The National Plan for Oral Health 1988–2002. Dar es Salaam, Tanzania: Ministry of Health and Social Welfare. 1988.

[22] Masanja IM, Mumghamba EGS. Knowledge on gingivitis and oral hygiene practices among secondary school adolescents in rural and urban Morogoro, Tanzania. Int J Dent Hyg. 2004;2(4):172–8. doi: 10.1111/j.1601-5037.2004.00096.x 16451492

[23] Simon ENM, Matee MI, Scheutz F. Oral health status of handicapped primary school pupils in Dar es Salaam, Tanzania. East Afr Med J. 2008;85(3):113–7. 18663883

[24] Ministry of Health. The United Republic of Tanzania. The fifth Tanzania National Oral Health Survey Report. 2020.

[25] National Academies of Sciences E, Division H and M, Services B on HC, Disabilities C on HCU and A with. Health-care utilization as a proxy in disability determination. National Academies Press. 2018. 29782136

[26] Schellenberg JA, Victora CG, Mushi A, de Savigny D, Schellenberg D, Mshinda H, et al. Inequities among the very poor: health care for children in rural southern Tanzania. Lancet. 2003;361(9357):561–6. doi: 10.1016/S0140-6736(03)12515-9 12598141

[27] Rossato MDS, Frítola M, Rossato PH, Francelino VCM, Poli-Frederico RC, Cardelli AAM, et al. Knowledge and Practices of Mothers towards Infant Oral Health Care in Their Children’s First Year of Life. J Health Scie. 2021;22(3):223–9. doi: 10.17921/2447-8938.2021v22n3p223-229

[28] Dieng S, Cisse D, Lombrail P, Azogui-Lévy S. Mothers’ oral health literacy and children’s oral health status in Pikine, Senegal: A pilot study. PLoS One. 2020;15(1):e0226876. doi: 10.1371/journal.pone.0226876 31971936 PMC6977722

[29] Linjawi AI, Bahaziq AM, Qari AH, Baeshen HA, Hassan AH. Impact of dental visits on oral health awareness in Saudi Arabia. JCDP. 2019. doi: 10.500 31597796

[30] Macek MD, Atchison KA, Chen H, Wells W, Haynes D, Parker RM, et al. Oral health conceptual knowledge and its relationships with oral health outcomes: Findings from a Multi-site Health Literacy Study. Community Dent Oral Epidemiol. 2017;45(4):323–9. doi: 10.1111/cdoe.12294 28271537 PMC5498245

[31] Junger ML, Griffin SO, Lesaja S, Espinoza L. Awareness Among US Adults of Dental Sealants for Caries Prevention. Prev Chronic Dis. 2019;16:E29. doi: 10.5888/pcd16.180398 30873938 PMC6429685

[32] Lawson DW, Borgerhoff Mulder M, Ghiselli ME, Ngadaya E, Ngowi B, Mfinanga SGM, et al. Ethnicity and child health in northern Tanzania: Maasai pastoralists are disadvantaged compared to neighbouring ethnic groups. PLoS One. 2014;9(10):e110447. doi: 10.1371/journal.pone.0110447 25353164 PMC4212918

[33] Butani Y, Weintraub JA, Barker JC. Oral health-related cultural beliefs for four racial/ethnic groups: Assessment of the literature. BMC Oral Health. 2008;8:26. doi: 10.1186/1472-6831-8-26 18793438 PMC2566974

[34] Mtuy TB, Mepukori J, Seeley J, Burton MJ, Lees S. The role of cultural safety and ethical space within postcolonial healthcare for Maasai in Tanzania. BMJ Glob Health. 2022;7(11):e009907. doi: 10.1136/bmjgh-2022-009907 36356986 PMC9660600

[35] Simangwa LD, Åstrøm AN, Johansson A, Minja IK, Johansson A-K. Oral diseases and oral health related behaviors in adolescents living in Maasai population areas of Tanzania: a cross-sectional study. BMC Pediatr. 2019;19(1):275. doi: 10.1186/s12887-019-1655-8 31391064 PMC6685221

[36] Okoroafor CC, Okobi OE, Owodeha-Ashaka M, Okobi E, Oluseye B, Ekpang OB, et al. Dental Health Knowledge Attitude and Practice Among University of Calabar Students. Cureus. 2023;15:e40055. doi: 10.7759/cureus.40055 PMC10325694 37425559

[37] Chen L, Hong J, Xiong D, Zhang L, Li Y, Huang S, et al. Are parents’ education levels associated with either their oral health knowledge or their children’s oral health behaviors? A survey of 8446 families in Wuhan. BMC Oral Health. 2020;20(1):203. doi: 10.1186/s12903-020-01186-4 32652985 PMC7353758

[38] Chinnakotla B, Susarla SM, Mohan DC, Turton B, Husby HM, Morales CP, et al. Associations between Maternal Education and Child Nutrition and Oral Health in an Indigenous Population in Ecuador. Int J Environ Res Public Health. 2022;20(1):473. doi: 10.3390/ijerph20010473 36612796 PMC9819843

[39] Minervini G, Franco R, Marrapodi MM, Di Blasio M, Ronsivalle V, Cicciù M. Children oral health and parents education status: a cross sectional study. BMC Oral Health. 2023;23(1):787. doi: 10.1186/s12903-023-03424-x 37875845 PMC10594879

[40] Levin KA, Davies CA, Topping GVA, Assaf AV, Pitts NB. Inequalities in dental caries of 5-year-old children in Scotland, 1993–2003. Eur J Public Health. 2009;19:337–42. 19307245 10.1093/eurpub/ckp035

[41] Badri P, Saltaji H, Flores-Mir C, Amin M. Factors affecting children’s adherence to regular dental attendance: a systematic review. J Am Dent Assoc. 2014;145(8):817–28. doi: 10.14219/jada.2014.49 25082930

[42] Alhareky M, Nazir MA. Dental Visits and Predictors of Regular Attendance Among Female Schoolchildren in Dammam, Saudi Arabia. Clin Cosmet Investig Dent. 2021;13:97–104. doi: 10.2147/CCIDE.S300108 33762854 PMC7982434

[43] Langevin SM, Michaud DS, Eliot M, Peters ES, McClean MD, Kelsey KT. Regular dental visits are associated with earlier stage at diagnosis for oral and pharyngeal cancer. Cancer Causes Control. 2012;23(11):1821–9. doi: 10.1007/s10552-012-0061-4 22961100 PMC3469776

[44] Levine Obe R, Stillman-Lowe C. Health education. Br Dent J. 2024;236(3):181–5. doi: 10.1038/s41415-024-7052-1 38332077

[45] Maasai Association. http://www.maasai-association.org/maasai.html

[46] J S D, Gupta AK, Veeri RB, Garapati P, Kumar R, Dhingra S, et al. Knowledge, attitude and practices towards visceral leishmaniasis among HIV patients: A cross-sectional study from Bihar, India. PLoS One. 2021;16(8):e0256239. doi: 10.1371/journal.pone.0256239 34404087 PMC8370793

[47] Yelland LN, Salter AB, Ryan P. Performance of the modified Poisson regression approach for estimating relative risks from clustered prospective data. Am J Epidemiol. 2011;174(8):984–92. doi: 10.1093/aje/kwr183 21841157

[48] Williamson T, Eliasziw M, Fick GH. Log-binomial models: exploring failed convergence. Emerg Themes Epidemiol. 2013;10(1):14. doi: 10.1186/1742-7622-10-14 24330636 PMC3909339

[49] Martinez BAF, Leotti VB, Silva G de SE, Nunes LN, Machado G, Corbellini LG. Odds Ratio or Prevalence Ratio? An Overview of Reported Statistical Methods and Appropriateness of Interpretations in Cross-sectional Studies with Dichotomous Outcomes in Veterinary Medicine. Front Vet Sci. 2017;4:193. doi: 10.3389/fvets.2017.00193 29177157 PMC5686058

[50] Wahengbam PP, Kshetrimayum N, Wahengbam BS, Nandkeoliar T, Lyngdoh D. Assessment of oral health knowledge, attitude and self-care practice among adolescents - a state wide cross-sectional study in Manipur, North Eastern India. J Clin Diagn Res. 2016;10:ZC65–70. 10.7860/JCDR/2016/20693.8002 PMC4963774 27504414

